# Communicable Animal Diseases1Read before the twenty-second annual meeting of the New Jersey State Sanitary Association at Lakewood, N. J., December 12, 1886.

**Published:** 1897-01

**Authors:** W. Herbert Lowe

**Affiliations:** Paterson, N. J., Formerly United States Veterinary Inspector of the Port of New York


### COMMUNICABLE ANIMAL DISEASES.[1]

By W. Herbert Lowe, D.V.S.,

PATERSON, N. J.,

FORMERLY UNITED STATES VETERINARY INSPECTOR OF THE PORT OF NEW YORK.

The importance of the subject of communicable and infectious diseases of animals can hardly be overestimated if we consider the money value of the animal industry of this State, including the intrinsic value of the animals themselves for their respective kinds

[1] Read before the twenty-second annual meeting of the New Jersey State Sanitary Association at Lakewood, N. J., December 12, 1886.

of service; the value of a wholesome and healthy meat-production and the large dairy interests within the confines of New Jersey. But the importance of the subject does not stop here. The economic is only one side of the question, for the public health and human life itself are dependent in no small degree on the physical condition and the healthfulness of our domestic animals.

Trichina, echinococcus, the beef and pork tapeworms, strongylus gigas, actinomycosis, and other parasites which affect man come directly from domestic animals. Glanders, rabies, anthrax, tuberculosis, and other deadly microbian diseases that affect the lower animals can also be communicated to the human family. The closest relationship and interdependence exist between communicable or infectious disease as they appear in animals and in man. Certain parasites make successive appearance in man and animals at different stages of their development, this being a condition of their propagation. The echinococcus, the beef and pork tapeworms, and even the trichina may be mentioned to illustrate this point. Our present knowledge would indicate that it is impossible for the echinococcus of the beef tænia to live in the same host or in a host of the same genus in both its larval and mature condition.

Man eats the meat and drinks the milk of the cow which may convey the seeds of consumption to himself, for the tubercle bacillus of the tuberculosis found in the cow is identical with the germ of the human being. Recent bacteriological work and discoveries have thrown great light upon the cause and mode of transmission of infectious diseases affecting men and animals. Bacteriological science is but in its infancy, but enough has been learned to settle many problems and to determine rational and practical methods in the crusade against microbian and other communicable diseases.

It is not long since we were engaged in the work of the suppression and extermination of pleuro-pneumonia contagiosa from this State and nation. It will be recalled that the disease had a foothold in certain counties of this State, and at one time bade fair to invade the whole country, threatening the animal industry of the nation, and entailing great money loss to a large part of the people. It did not take long for the people to reach legislative halls and secure proper State and national legislation for the suppression and extermination of the plague. New Jersey is so situated geographically and the animal traffic so extensive that the work of extermination became no trifling matter. Our State Board of Health was ever diligent in stamping out new outbreaks and in limiting extensions of the disease, but it was practically impossible to prevent fresh introductions of the contagion without Federal aid, which was not withheld. The work of extermination of contagious pleuro-pneumonia soon became a national question, and the professional talent and the executive ability of that distinguished veterinarian, Dr. D. E. Salmon, the Chief of the United States Bureau of Animal Industry, were equal to the occasion, and the animal wealth of the nation has been protected and that dreaded plague exterminated in the United States.

I have alluded hastily to these facts connected with the extermination of pleuro-pneumonia contagiosa in the United States to show that the people of this country are not slow to protect the wealth of the nation from an animal plague. In the matter of contagious pleuro-pneumonia it was solely a financial question, as above indicated, for no one ever heard of a human being contracting bovine contagious pleuro-pneumonia; whereas in the case of many of the other contagious animal diseases the health and the very lives of the people are endangered if proper sanitary and limiting measures are not adopted, and if safeguards are not heeded regarding the meat- and milk-supply. It is an old saying "that health is better than wealth," but it seems to me that the truth of this saying is sometimes lost sight of by our people in their enterprise and the multiplicity of their affairs.

(To be continued.)

---

Veterinarian Meyer, of New South Wales, in the *Breeders' Gazette*, recommends the following drench for stomach or intestinal worms in sheep: Arsenic, one ounce; potash or soda, two ounces; boiled together slowly for one hour in one gallon of water and stirred occasionally; then when cold strained and water added to make four gallons. Weaners, one fluidounce; adult sheep, one and one-half fluidounces at a dose. In aggravated cases these doses may be increased a half-ounce. Keep the sheep from water until midday, drinking water immediately afterward is apt to produce fatal results.

Canada sheep in bond may now be exported from Portland, Me., through arrangements with the Department of Agriculture, and cattle and sheep from Boston after proper examination at Island Pond and St. Albans, Vermont.

Veterinarian J. E. Rickards, in the *Veterinary Record*, highly extols carbonate of bismuth and bicarbonate of soda in cases of jaundice in canine practice. One part of the bismuth to two parts of the soda.

